# Recommendations Regarding Tobacco Use and Secondhand Smoke Exposure from the Community Preventive Services Task Force

**Published:** 2013-06-14

The Community Preventive Services Task Force recently posted new information about two recommendations: 1) “Reducing Tobacco Use and Secondhand Smoke Exposure: Reducing Out-of-Pocket Costs for Evidence-Based Tobacco Cessation Treatments,” available at http://www.thecommunityguide.org/tobacco/outofpocketcosts.html, and 2) “Reducing Tobacco Use and Secondhand Smoke Exposure: Quitline Interventions,” available at http://www.thecommunityguide.org/tobacco/quitlines.html.

Established in 1996 by the U.S. Department of Health and Human Services, the task force is an independent, nonfederal, unpaid panel of public health and prevention experts whose members are appointed by the Director of CDC. The task force provides information for a wide range of decision makers on programs, services, and policies aimed at improving population health. Although CDC provides administrative, research, and technical support for the task force, the recommendations developed are those of the task force and do not undergo review or approval by CDC.

